# Emergence, spread and characterisation of the SARS-CoV-2 variant B.1.640 circulating in France, October 2021 to February 2022

**DOI:** 10.2807/1560-7917.ES.2023.28.22.2200671

**Published:** 2023-06-01

**Authors:** Gwenola Picard, Lucie Fournier, Anna Maisa, Claire Grolhier, Souhaila Chent, Caroline Huchet-Kervalla, Jeanne Sudour, Maël Pretet, Laurence Josset, Sylvie Behillil, Justine Schaeffer, Laboratory group, COVID-19 Investigation Group, Members of the Laboratory group, Laura Verdurme, Anaïs Soares, Hugues Leroy, Mélanie Jimenez-Pocquet, Christophe Rodriguez, Tanguy Martin-Denavit, Pierre-Edouard Fournier, Stephan Kemeny, Aurélie Guigon, Vincent Thibault, Audrey Mirand, Members of the COVID-19 Investigation group, Nathalie Thomas, Mohamed Hamidouche, Leila Bekheira, Anais Lamy, Anna Lloyd, Alice Brembilla, Michée Géraud Vikpognon, Adeline Riondel, Alizé Mercier, Alain LeTertre

**Affiliations:** 1 Bretagne Regional Office, Direction des Régions (DiRe), Santé publique France, Rennes, France; 2 Direction des Maladies Infectieuses (DMI), Santé publique France, Saint-Maurice, France; 3 Department of Virology, INSERM, IRSET UMR-S 1085, Pontchaillou University Hospital, Université de Rennes, Rennes, France; 4 Hauts-de-France Regional Office, Direction des Régions (DiRe), Santé publique France, Lille, France; 5 Pays-de-la-Loire Regional Office, Direction des Régions (DiRe), Santé publique France, Nantes, France; 6 Direction DATA, Santé publique France, Saint-Maurice, France; 7 Centre National de Référence Virus des Infections Respiratoires (dont la grippe) and Plateforme GENEPII Laboratoire de Virologie des HCL, Hopital de la Croix Rousse, Lyon, France; 8 Laboratoire Virpath, CIRI, Inserm U1111, CNRS UMR 5308, ENS de Lyon, UCBL, Lyon, France; 9 Centre National de Référence Virus des Infections Respiratoires (dont la grippe), Institut Pasteur, Paris, France; 10 Unité de Génétique Moléculaire des Virus à ARN - UMR3569 CNRS, Université de Paris, Paris, France; 11 Members of the Laboratory group are listed under Collaborators; 12 Members of the COVID-19 Investigation group are listed under Collaborators

**Keywords:** COVID-19, SARS-CoV-2, whole genome sequencing, genomic surveillance, emerging variant, B.1.640

## Abstract

**Background:**

Successive epidemic waves of COVID-19 illustrated the potential of SARS-CoV-2 variants to reshape the pandemic. Detecting and characterising emerging variants is essential to evaluate their public health impact and guide implementation of adapted control measures.

**Aim:**

To describe the detection of emerging variant, B.1.640, in France through genomic surveillance and present investigations performed to inform public health decisions.

**Methods:**

Identification and monitoring of SARS-CoV-2 variant B.1.640 was achieved through the French genomic surveillance system, producing 1,009 sequences. Additional investigation of 272 B.1.640-infected cases was performed between October 2021 and January 2022 using a standardised questionnaire and comparing with Omicron variant-infected cases.

**Results:**

B.1.640 was identified in early October 2021 in a school cluster in Bretagne, later spreading throughout France. B.1.640 was detected at low levels at the end of SARS-CoV-2 Delta variant’s dominance and progressively disappeared after the emergence of the Omicron (BA.1) variant. A high proportion of investigated B.1.640 cases were children aged under 14 (14%) and people over 60 (27%) years, because of large clusters in these age groups. B.1.640 cases reported previous SARS-CoV-2 infection (4%), anosmia (32%) and ageusia (34%), consistent with data on pre-Omicron SARS-CoV-2 variants. Eight percent of investigated B.1.640 cases were hospitalised, with an overrepresentation of individuals aged over 60 years and with risk factors.

**Conclusion:**

Even though B.1.640 did not outcompete the Delta variant, its importation and continuous low-level spread raised concerns regarding its public health impact. The investigations informed public health decisions during the time that B.1.640 was circulating.

**Key public health message**

**What did you want to address in this study?**

When a new SARS-CoV-2 variant appears, it is difficult to predict if it will have an increased impact compared with previously circulating variants. In our study, we describe the detection of an emerging variant in France in autumn 2021, named B.1.640, and the investigations conducted to characterise it. The final objective was to evaluate whether the spread of B.1.640 would justify implementation of specific control measures.

**What have we learnt from this study?**

Our data show that B.1.640 was able to circulate when the Delta variant was dominant but progressively disappeared during the emergence of Omicron in late 2021. Symptoms and severity of a B.1.640 infection was similar to the Delta variant. Hospitalised B.1.640-infected cases were primarily older people (≥ 60 years) and individuals with risk factors, who would have been at risk of severe COVID-19 with any variant.

**What are the implications of your findings for public health?**

Our findings illustrate the complementarity of the different strategies in place in France to rapidly identify, investigate, characterise and evaluate unknown SARS-CoV-2 variants. B.1.640 emergence did not justify the implementation of specific control measures, as it circulated at low levels and did not show an increased disease severity.

## Introduction

Since the emergence of severe acute respiratory syndrome coronavirus 2 (SARS-CoV-2), over 650 million cases have been detected worldwide by the end of 2022 [1]. Despite its lower mutation rate compared with other RNA viruses, genetic divergence of SARS-CoV-2 has led to the definition of lineages and sub-lineages [2,3]. Lineage classification, using the Pango (Phylogenetic Assignment of Named Global Outbreak) nomenclature, is a very detailed naming system aimed at following viral evolution at a large scale, and not a tool designed for public health purposes [4]. The emergence of a new lineage might but does not necessarily have an effect on viral characteristics, e.g. transmissibility, immune escape or severity, and therefore on its public health impact. For the purpose of public health, it is of major importance to discriminate between neutral and high-effect genetic changes and identify those that impact transmissibility, severity, immune escape, efficiency of treatments and diagnostic methods [5].

In the first months of the COVID-19 pandemic in spring 2020, no major genetic changes were observed in the circulating SARS-CoV-2 strains (referred to as ‘wild-type’). The Alpha variant (Pango lineage designation B.1.1.7) of SARS-CoV-2 , detected in September 2020, was the first SARS-CoV-2 lineage for which a major change in characteristics was documented. The Alpha variant showed an increased transmissibility compared with previously circulating strains and progressively replaced those strains in Europe and other parts of the world [6]. Following the emergence of the Alpha variant, a classification system for SARS-CoV-2 variants was designed by the World Health Organization (WHO): variant of concern (VOC), variant of interest (VOI) and variant under monitoring (VUM) [7]. Unlike Pango lineage definitions, which aim at classifying SARS-CoV-2 strains according to their genetic similarities, variants are classified by the WHO as VOC/VOI/VUM according to their phenotypic characteristics and their public health impact. SARS-CoV-2 variants that were later designated VOCs include Delta (Pango lineage designation B.1.617.2, October 2020) and Omicron (Pango lineage designation B.1.1.529, November 2021).

On 15 October 2021 in Bretagne, France, the Lorient South Bretagne hospital group informed the Bretagne Regional Health Agencies (ARS) and the regional offices of Santé publique France (French National Public Health agency, SpF) of four confirmed SARS-CoV-2 infections with an unusual mutation screening result: a wild-type L452 residue on the spike protein at a time when the Delta variant (carrying the mutation L452R) was dominant in France. Whole genome sequencing (WGS) identified this a new SARS-CoV-2 variant, which was assigned to lineage B.1.640 in late November 2021.

In this study, we describe the detection and spread of the SARS-CoV-2 B.1.640 variant in the French population. Additional investigations of B.1.640-infected cases were conducted to assess the characteristics of this new variant and better evaluate its public health impact.

## Methods

### SARS-CoV-2 variant surveillance

In France in 2021–22, SARS-CoV-2 testing by antigen test or RT-qPCR was available for free to the general population. The surveillance of SARS-CoV-2 variants includes two systems: identification of a predefined set of target mutations using multiplex RT-qPCR (referred to in this manuscript as ‘mutation screening’ and systematically performed for samples positive for SARS-CoV-2 by RT-qPCR) and genomic surveillance (using Sanger sequencing or WGS, referred to in this manuscript as ‘sequencing’).

Genomic surveillance of SARS-CoV-2 is coordinated by SpF and the National Agency for Research on AIDS and Viral Hepatitis and Emerging Infectious Diseases (ANRS|MIE) through the consortium for surveillance and research on EMERging infectious diseases using microbial GENomics (EMERGEN) [8]. This consortium comprises: four national public platforms, including two laboratories of the National Reference Centre (NRC) for respiratory infection viruses and two NRC expert laboratories; four national private platforms; and 47 other French laboratories performing SARS-CoV-2 sequencing. ‘Flash surveys’ were designed for representative random genomic surveillance: every week, a pre-set proportion of samples positive for SARS-CoV-2 (defined according to European Centre for Disease Prevention and Control (ECDC) guidelines) were sequenced [9]. Representative sequencing is complemented by targeted sequencing (e.g. reinfections, vaccine failure, severe cases and travellers) and interventional sequencing (e.g. outbreak investigation, specific situation at local level).

All sequencing results produced by the consortium are submitted to national (EMERGEN-database (DB)) and international (GISAID) databases. Sequences uploaded to EMERGEN-DB are regularly reanalysed to account for changes in the Nextstrain (clade) and Pango lineage (lineage) nomenclatures [10,11]. For this study, final variant assignation was performed using Nextclade version 1.10.2 [10] and Pango lineage version 3.1.20 [4].

### Case definition

The B.1.640 variant was defined from sequencing data using the Pango lineage nomenclature or mutation proxy S:P9L, S:E96Q, S:R346S, S:Y449N, S:P681H and S:T859N in the output of the Nextclade analysis tool [10,12]. Confirmed B.1.640 cases were defined as patients with a positive SARS-CoV-2 test and a sequencing result demonstrating infection by B.1.640 between week 41 2021 and week 2 2022 in France.

### Case investigation

Sequencing laboratories shared contact details of B.1.640 confirmed cases with the SpF regional office of their residence using a secured messaging system. SpF regional offices contacted the cases, explained the context of the investigation and, upon consent, interviewed them using a standardised questionnaire. Data were collected on demographics (age, sex), travel history (travel dates, countries visited), clinical presentation (presence/absence; date of symptom onset; asthenia/fatigue, cough, fever, myalgia, headache, runny nose, ageusia, sore throat, anosmia, fever feeling, shortness of breath, diarrhoea, nausea/vomiting, dyspnoea, cold, influenza-like syndrome, dizziness, acute respiratory distress syndrome (ARDS), abnormal lung auscultation (ALA) or other) and outcome (hospitalisation and intensive care (ICU) admissions, dates of admission and discharge, death), risk factors (hypertension, obesity, diabetes, chronic respiratory disease, renal insufficiency, cancer, immunosuppression, liver disease, heart disease, neuromuscular pathology, pregnancy or other), previous SARS-CoV-2 infection and vaccination status (number of doses and date of administration). In case of hospitalisation, the attending physicians were interviewed. 

A similar investigation was performed during the emergence of the Omicron variant (mostly BA.1) in France and was used for comparison [13]. 

‘Investigated cases’ were defined as confirmed cases that were interviewed. Anonymised questionnaires were shared with the SpF team in charge of the analysis.

### Statistical analysis and visualisation

Statistical analyses were performed using R (version 4.0.5) [14]. Epidemiological data of B.1.640 and Omicron cases were compared by univariate analysis (chi-squared tests and odds ratios (OR)). Variables significantly associated with hospitalisation in the univariate analysis (p < 0.05) were included in a Poisson regression model. All combinations of these variables were tested and the model with the lowest Akaike Information Criterion was used as a final model. Graphs were created using R (package ggplot2) [15].

## Results

### Cluster investigation and identification of B.1.640

Following the initial alert from Bretagne in October 2021, an investigation was conducted by epidemiologists at the regional office of SpF and the ARS. Patients with a non-Delta mutation screening result (wild type L452) were contacted to investigate transmission chains and possible importations. The primary case was identified and had recently travelled to Congo (Brazzaville). Upon returning, the primary case visited relatives in Bretagne, whose children later tested positive for SARS-CoV-2. These children attended the primary school where 22 children and one adult also tested positive for SARS-CoV-2.

**Figure 1:**
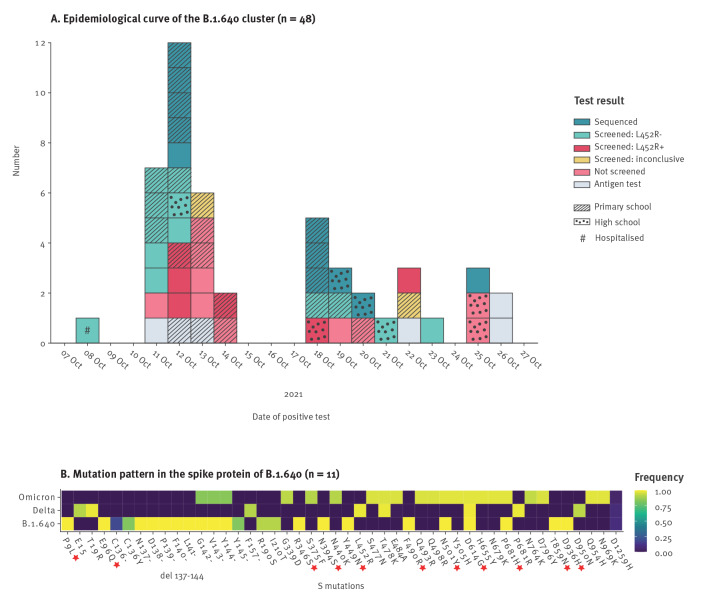
Detection and description of the SARS-CoV-2 variant B.1.640, France, October 2021

Overall, this cluster included 48 confirmed SARS-CoV-2 cases identified between 8 and 26 October 2021 (Figure 1A). Samples from 11 cases were sequenced and mutation screening was performed for 21, with screening results L452R− (n = 13), L452R+ (n = 6) and inconclusive (n = 2). Of the remaining 16 (with no mutation screening), 10 were tested by RT-qPCR and six by antigen tests. Mass testing was performed in individuals around this cluster (i.e. family, school and community contacts) and all 521 samples were negative by RT-qPCR. Cases were detected in a primary school (n = 23 cases) and a high school (n = 7 cases) (Figure 1A). The sex ratio among cases was 1. The mean age of adults was 47 years (range: 19–88). At the time of the investigation, primary school children were not eligible for COVID-19 vaccination. Among the eligible population (> 12 years of age), 10 of 26 cases were fully vaccinated. Only one case was hospitalised in the ICU.

SARS-CoV-2: severe acute respiratory syndrome coronavirus 2.

A. Epidemiological curve of the B.1.640 cluster in Bretagne (n = 48), according to sampling date of the first SARS-CoV-2 positive test. All cases tested positive by antigen or RT-qPCR test. Further characterisation was performed by mutation screening with RT-qPCR targeting spike mutation L452R or whole genome sequencing, as indicating by the filling colour. Location where cases were likely infected (primary school or high school) are indicated.

B. Mutation pattern in the spike protein of B.1.640 compared with Delta and Omicron sequences. This heatmap includes the mutations detected in at least one of the B.1.640 sequences from the cluster (n = 11) from Bretagne, or at least 90% of Delta (n = 5,826) or Omicron (n = 2,568) sequences detected in Bretagne before 14 March 2022 (ambiguous positions counted as wild type). Red stars indicate mutations initially used to characterise B.1.640.

Whole genome sequencing was performed for 11 cases of this cluster. A new SARS-CoV-2 variant was identified and assigned to lineage B.1.640. At the time of the cluster investigation, similar sequences from Congo were available in the international sequencing database GISAID (https://www.gisaid.org). The B.1.640 variant exhibited an unusual pattern of mutations in the spike protein: P9L, E96Q, R346S, N394S, Y449N, F490R, N501Y, D614G, P681H, T859N and D936H (Figure 1B) [16]. A large deletion in the N-terminal domain (positions 136–144) was also found in all 11 sequences.

### Circulation of the B.1.640 variant in France

**Figure 2:**
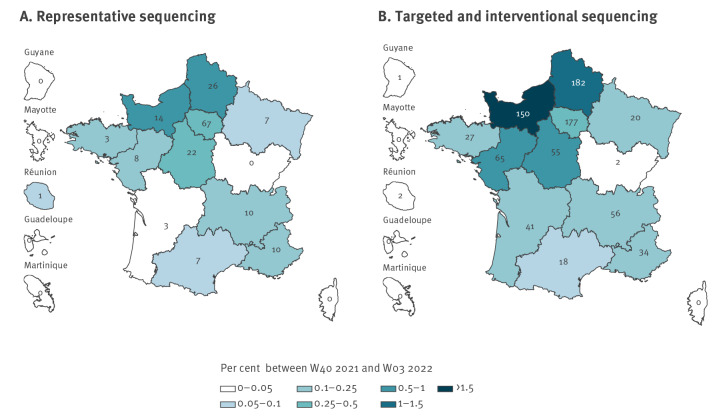
Geographical distribution of B.1.640 variant sequences from representative sequencing (n = 179^a^) or targeted and interventional sequencing (n = 830), France, week 40 2021−week 3 2022

^a^ One sequence from representative surveillance had missing data on the region, so only 178 are shown.

Following its first detection in October 2021, B.1.640 circulated in France until early 2022. Representative genomic surveillance data (Flash surveys) indicated that B.1.640 circulated at low levels (with a maximum of 0.52%) from week 41 2021 to week 2 2022 (Table 1). This period overlapped with the end of the period when the Delta variant was dominant and the emergence of the Omicron variant (sub-lineage BA.1). B.1.640 was detected through representative genomic surveillance in 11 of 13 regions of metropolitan France and Réunion (Figure 2A). Regions with the highest B.1.640 circulation were Hauts-de-France and Normandie.

**Table 1:** Proportion of SARS-CoV-2 variants detected by representative genomic surveillance, during the circulation of the B.1.640 variant, France, week 40 2021−week 3 2022

Sampling date	SARS-CoV-2 variant							
	B.1.640		Delta		Omicron		Other	
	n	%	n	%	n	%	n	%
Week 40 2021	0	0	1,376	99.93	0	0	1	0.07
Week 41 2021	1	0.06	1,788	99.89	0	0	1	0.06
Week 42 2021	2	0.05	3,808	99.95	0	0	0	0
Week 43 2021	8	0.10	8,295	99.88	0	0	2	0.02
Week 44 2021	21	0.28	7,447	99.72	0	0	0	0
Week 45 2021	10	0.16	6,386	99.83	0	0	1	0.02
Week 46 2021	9	0.11	7,995	99.88	0	0	1	0.01
Week 47 2021	19	0.34	5,513	99.57	4	0.07	1	0.02
Week 48 2021	27	0.41	6,526	99.33	15	0.23	2	0.03
Week 49 2021	26	0.49	5,182	98.14	71	1.34	1	0.02
Week 50 2021	27	0.52	4,662	90.12	484	9.36	0	0
Week 51 2021	8	0.18	2,629	59.60	1,771	40.15	3	0.07
Week 52 2021	15	0.19	2,680	33.45	5,316	66.34	2	0.02
Week 1 2022	3	0.07	684	15.38	3,753	84.41	6	0.13
Week 2 2022	3	0.06	299	5.86	4,797	94.02	3	0.06
Week 3 2022	0	0	143	2.72	5,113	97.26	1	0.02
**Total**	**179**	**0.21**	**65,413**	**75.24**	**21,324**	**24.53**	**25**	**0.03**

SARS-CoV-2: severe acute respiratory syndrome coronavirus 2.

The maps show B.1.640 cases by region of residence of the case. Colours indicate the percentage during the sampling period and numbers are the absolute number of sequences per region. Of note, Guadeloupe, Martinique, Réunion, Mayotte and Guyane are overseas territories.

Through representative genomic surveillance, 179 B.1.640 sequences were detected. The total number of B.1.640 cases identified in France was much higher, with 1,009 sequences (see Supplementary Table S1 for time distribution of B.1.640 sequences (total and from representative sequencing)). Most of these additional sequences were obtained through systematic interventional sequencing of samples with mutation screening L452R−, which was implemented at the emergence of Omicron. The B.1.640 variant was detected through targeted and interventional sequencing in 12 of 13 regions of metropolitan France, and overseas territories Réunion and Guyane, with the most cases in Hauts-de-France and Normandie (Figure 2B).

### Demographic characteristics of investigated B.1.640 cases

**Figure 3:**
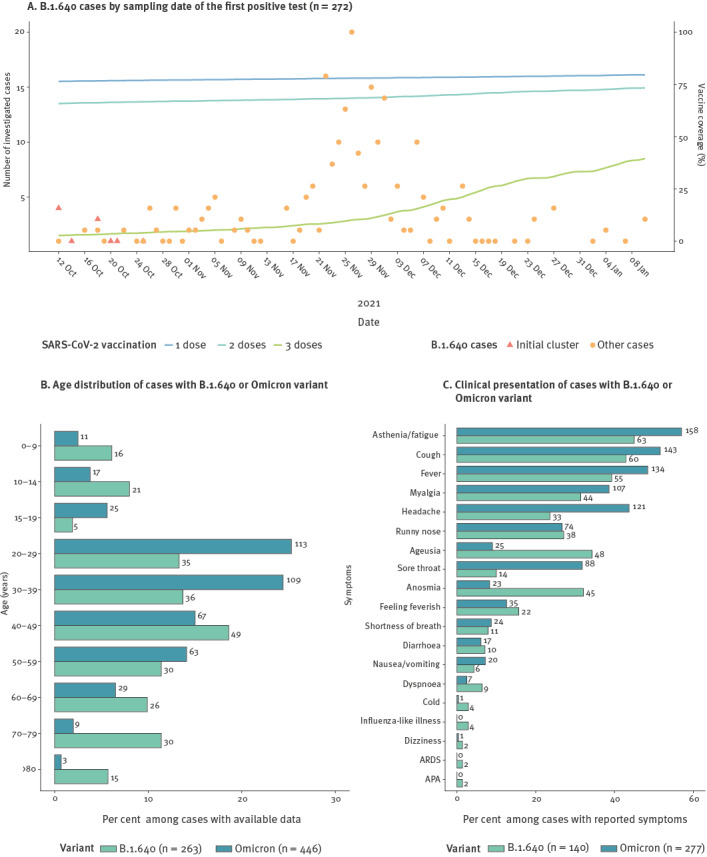
Characteristics of investigated B.1.640 cases, France, October 2021–January 2022 (n = 272)

Overall, 272 B.1.640 cases confirmed by sequencing were investigated by the regional offices of SpF in collaboration with the ARS. Sampling dates ranged from 12 October 2021 to 10 January 2022 (Figure 3A). Seventy-four percent of investigated cases had a sampling date between 15 November 2021 and 19 December 2021, which corresponded to the period with systematic sequencing of L452R− samples (Figure 3A). See Supplementary Table S1 for time distribution of investigated cases. Omicron cases, previously published [13] and used in this study as a reference (data can be found in Supplementary Table S1), were investigated during the same period.

A. Red triangles indicate cases from the initial cluster in Bretagne (n = 48) and orange circles other investigated cases (n = 224). The blue and green lines indicate vaccine coverage in the French population over the study period.

B and C. Age distribution and clinical presentation of investigated cases infected by B.1.640 or Omicron. Bars correspond to the percentage of each category among cases with available data. Number of cases are indicated next to the corresponding bars.

Among the investigated B.1.640 cases, the male/female sex ratio was 1 and median age was 42 (interquartile range (IQR): 26–62). This was similar to the sex ratio and median age of all sequenced B.1.640 cases (sex ratio: 1, median age: 41, IQR: 26–60) and all cases sequenced through representative surveillance (sex ratio: 1.1, median age: 42, IQR: 28–57), but significantly different from investigated Omicron cases (sex ratio: 0.8, median age: 35 IQR: 25–48, Wilcoxon signed-rank test p < 0.0001). The overall age distribution was different between investigated B.1.640 and Omicron cases, with a higher proportion of children below 14 years of age (14% for B.1.640 vs 6% for Omicron) and people aged over 60 years (27% for B.1.640 vs 9% for Omicron) (Figure 3B). See Supplementary Table S2 for detailed characteristics of B.1.640 and Omicron cases. These differences can be explained by large B.1.640 clusters including either children (19% of cases from clusters were below 14 years old vs 10% excluding clusters) or older people (32% of cases from clusters were over 60 years old vs 21% excluding clusters). See Supplementary Figure S1 for age distribution of cases belonging to clusters or not.

### Distribution and transmission patterns

Eleven regions of metropolitan France and Réunion investigated B.1.640 cases, with a higher number of cases from Hauts-de-France (n = 64), Normandie (n = 52) and Bretagne (n = 35) (Table 2). In these three regions, large clusters of B.1.640 were identified and investigated. The previously described cluster in Bretagne accounted for 11 investigated cases and another smaller cluster for three investigated cases. Twenty-two of the investigated cases from Normandie belonged to a cluster of 75 cases that was previously published [17]. A hospital cluster identified in October 2021 in Hauts-de-France accounted for 18 of 64 investigated cases in this region. A total of nine patients, 13 healthcare workers (HCW) and 4 HCW family members were identified in two distinct hospital departments. Four deaths were reported but only one was due to COVID-19. Overall, 48% of investigated B.1.640 cases with available data (115/242) were associated with clusters larger than their household (Table 2).

**Table 2:** Demographic and clinical characteristics of investigated B.1.640 (n = 272) and Omicron (n = 469) variant cases, France, October 2021–January 2022

Characteristics^a^		SARS-CoV-2 variant			
		B.1.640		Omicron	
		n	%	n	%
**Sex^b^** (B.1.640 = 205; Omicron = 358)	Female	101	49	196	55
	Male	104	51	162	45
**Region** (B.1.640 = 272; Omicron = 469)	Auvergne-Rhône-Alpes	13	5	42	9
	Bourgogne-Franche-Comté	0	0	6	1
	Bretagne	35	13	27	6
	Centre-Val de Loire	8	3	40	9
	Corse	0	0	31	7
	Grand Est	9	3	29	6
	Guadeloupe	0	0	25	5
	Guyane	0	0	1	0
	Hauts-de-France	64	24	20	4
	Île-de-France	32	12	105	22
	Réunion	1	< 1	19	4
	Martinique	0	0	20	4
	Normandie	52	19	23	5
	Nouvelle-Aquitaine	13	5	10	2
	Occitanie	7	3	21	4
	Pays de la Loire	22	8	12	3
	Provence-Alpes-Côte d’Azur	16	6	38	8
**Travel history^c^** (B.1.640 = 237; Omicron = 408)	Yes	34	14	149	37
	No	203	86	259	63
**Cluster^d^** (B.1.640 = 242; Omicron = 297)	Yes	115	48	84	28
	No	127	52	213	72
**Symptomatic infection** (B.1.640 = 240; Omicron = 422)	Yes	207	86	376	89
	No	33	14	46	11
**Risk factors^e^** (B.1.640 = 116; Omicron = 284)	Yes	29	25	45	16
	No	87	75	239	84
**Hospitalisation** (B.1.640 = 226; Omicron = 294)	Yes	19	8	7	2
	No	207	92	287	98
**Intensive care unit** (B.1.640 = 189; Omicron = 292)	Yes	5	3	0	0
	No	184	97	292	100
**Previous SARS-CoV-2 infection** (B.1.640 = 102; Omicron = 279)	Yes	4	4	39	14
	No	98	96	240	86
**Vaccination status** (B.1.640 = 217; Omicron = 412)	One dose	6	3	20	5
	Two doses	126	58	251	61
	Three doses	19	9	28	7
	Not eligible (< 12 years old)	28	13	24	6
	Unvaccinated	38	18	89	22

SARS-CoV-2: severe acute respiratory syndrome coronavirus 2.

^a^ For each category, percentages were calculated for the number of cases with available data. Respective denominators for B.1.640 and Omicron variants are indicated in brackets.

^b^ Sex was recorded as a binary variable. Other or no answer were recorded as ‘missing data’.

^c^ Travel or contact with a person who travelled in the last 14 days.

^d^ Clusters larger than their household.

^e^ Hypertension, obesity, diabetes, chronic respiratory disease, renal insufficiency, cancer, immunosuppression, liver disease, heart disease, neuromuscular pathology, pregnancy or other.

Of note, Guadeloupe, Martinique, Réunion, Mayotte and Guyane are overseas territories.

Travel or contact with a person who travelled in the last 14 days was reported by 34 (14%) investigated B.1.640 cases. Twelve cases associated with travel from Congo were identified between week 41 2021 and week 2 2022.

### Immune status

Four investigated B.1.640 cases reported a previous SARS-CoV-2 infection (4%, Table 2), which was significantly lower than investigated Omicron cases (14%, p = 0.046, see Supplementary Table S2 for detailed characteristics of B.1.640 and Omicron cases). Vaccination status was available for 217 investigated B.1.640 cases. Twenty-eight cases below the age of 12 years (13%) were excluded, as this age group was not eligible for vaccination at the time of the investigation. Unvaccinated cases represented 18% of B.1.640 cases, and among those vaccinated, 3% had received one dose, 58% had received two doses and 9% had received three doses (Table 2). Over the study period, the proportion of the general population vaccinated with one, two and three dose(s) increased from 76% to 80%, 65% to 73% and 2 to 30%, respectively (Figure 3A). The median delay between the last vaccine dose received and the first positive test was 149 days (IQR: 104–178).

### Clinical presentation

Of 240 investigated B.1.640 cases for which the information was available, 207 (86%) had a symptomatic infection (Table 2). Of the 140 cases reporting specific symptoms, the most common were asthenia (45%), cough (43%), fever (39%), ageusia (34%), anosmia (32%) and myalgia (31%) (Figure 3C). Compared with investigated Omicron cases, B.1.640 cases had a higher rate of ageusia (OR: 4.77, 95% confidence interval (CI): 2.73–8.52, p < 0.0001) and anosmia (OR: 4.86, 95% CI: 2.74–8.83, p < 0.0001). Severe symptoms, such as dyspnoea, APA and ARDS were also reported by a higher proportion of investigated B.1.640 cases (6%, 1.4% and 1.4% for B.1.640 vs 2.5%, 0% and 0% for Omicron, respectively) (Figure 3C). Only nine investigated B.1.640 cases presented with these three more severe symptoms. They were all younger than 70 years old (median age: 39, IQR: 30–49) and two reported risk factors.

Overall, 19 (8%) investigated B.1.640 cases were hospitalised and five (3%) were admitted to the ICU (Table 2). Among hospitalised cases, four were hospitalised for another reason than COVID-19. Hospitalisation and ICU admission rates were higher than for Omicron cases, among which seven hospitalisations (2%, p < 0.001) and no ICU admission (p < 0.05) were reported (see Supplementary Table S2 for detailed characteristics of B.1.640 and Omicron cases). Median age of hospitalised B.1.640 cases was 75 years (IQR: 59–84). Eight hospitalised cases (44%) were over 80 years old, which is more than for non-hospitalised cases (3%, p < 0.0001). Risk factors were more frequent among hospitalised B.1.640 cases (71% vs 22%, p < 0.05). See Supplementary Table S3 for detailed characteristics of hospitalised vs non-hospitalised B.1.640 cases. For hospitalised B.1.640 cases with available vaccination status, 5 of 10 were unvaccinated.

Univariate analysis of both B.1.640 and Omicron investigated cases showed that factors associated with hospitalisation were B.1.640 infection (compared with Omicron, OR: 4.4, 95% CI: 1.8–10.6), age over 80 years (OR: 30.3, 95% CI: 10.2–90.3) and presenting risk factors (OR: 8.7, 95% CI 2.5–29.7). COVID-19 vaccination with two or three doses was associated with lower hospitalisation rates (OR: 0.29, 95% CI: 0.11–0.75). After adjusting for age (≥ 80 vs < 80 years) and vaccination status (2–3 doses vs 0–1 dose) using Poisson regression, B.1.640 infection was no longer associated with hospitalisations (adjusted risk ratio: 2.35, 95% CI: 0.671–9.28). See Supplementary Table S4 for parameters of the multivariate model. Cases admitted to the ICU had a median age similar to all hospitalised cases (74 years, IQR: 42–75). One case hospitalised for another reason than COVID-19 died in the hospital.

## Discussion

We describe the emergence of a new variant, B.1.640, in France, which was detected and monitored through genomic surveillance. In this situation, we used targeted/interventional sequencing to identify and investigate specific signals, and representative sequencing to provide a more global picture. The B.1.640 variant was first detected in the Bretagne region, with both epidemiological and genomic investigations pointing to an importation from Congo. The B.1.640 variant then spread throughout the country, most likely because of several introductions. The stable circulation of the B.1.640 variant in France, over both a low-incidence period and an epidemic wave, suggested a strong competitiveness compared with Delta. However, the B.1.640 variant did not expand to the same extent in other countries, with 80% of B.1.640 sequences available on GISAID originating from France. The low number of sequences from Central African countries can be explained by their limited sequencing capacities; however, neighbouring European countries have more similar genomic surveillance systems to France. The difference of spread of the B.1.640 variant between France and other European countries illustrates the impact of founder effects, i.e. the number of introductions and their association with large clusters or super-spreader events, on the dynamics of emerging variants [18]. A similar example would be the Delta variant sub-lineage AY.4.2, which largely circulated in the United Kingdom but remained at low levels in other countries [19].

A mutational profile of B.1.640 was available soon after its detection in France, which guided a first evaluation of this variant. The spike substitution N501Y was detected in December 2020 with the emergence of the Alpha variant [20]. This mutation increases affinity between the spike protein and its receptor ACE2, and was associated with the higher transmissibility of the Alpha, Beta (Pango lineage designation B.1.351) and Gamma (Pango lineage designation P.1) [21-23]. Substitution P681H is located near the furin cleavage site of the spike protein. Mutations at this site have been shown to increase fusogenicity in the Alpha, Gamma and Delta variants and could contribute to their higher infectiveness [24,25]. Finally, the B.1.640 variant frequently showed a large deletion in the N-terminal domain of the spike (positions 136–144), which had not been previously described. This domain is an important target for neutralising antibodies and, therefore, this deletion could impact the effectiveness of post-vaccination or post-infection humoral responses [26]. Mutations found in the B.1.640 variant raised concern about its possible public health impact and led to its classification as a VUM. However, knowledge on individual mutations was not sufficient to extrapolate its characteristics because of potential antagonistic and/or synergistic effects. Additional investigations were performed and seroneutralisation assays showed an increased immune escape of B.1.640 compared with the Delta variant, justifying the classification of B.1.640 as a VOI in France [27].

Investigations of B.1.640 cases were performed in order to complement genetic and in vitro data. Children (≤ 14 years) and older people (≥ 60 years) were over-represented in investigated B.1.640 cases because of large clusters in these age groups. The proportion of unvaccinated individuals was higher in B.1.640 cases (18%) compared to the population aged above 12 years at the time of the study (13% on 5 October 2021 and decreasing since), suggesting some effectiveness of vaccination against B.1.640 infection. The proportion of cases vaccinated with three doses was difficult to interpret, as the overall vaccine coverage for the third dose rapidly increased from 2% to 30% over the study period. Previous SARS-CoV-2 infection was reported by 4% of B.1.640 cases, which was similar to previous studies on Delta but much lower than with Omicron [28]. Anosmia and ageusia were more frequently found in B.1.640 cases compared with Omicron cases, which is consistent with the clinical presentation of pre-Omicron SARS-CoV-2 variants [29]. Eight percent of investigated B.1.640 cases were hospitalised, which was higher than reported for investigated Omicron cases. However, this difference seemed to be caused by the over-representation of cases over 80 years of age and presenting risk factors among B.1.640 cases. It is important to note that the small sample size of the study would only allow the detection of significant differences, especially when adjusting for several variables.

Our study design has important limitations. The concomitance of our investigations with enhanced surveillance of non-Delta cases during the emergence of the Omicron variant allowed us to detect more B.1.640 cases. However, this peak of detection did not correspond to an increased circulation and only cases sequenced through representative surveillance must be considered when monitoring B.1.640 circulation. In addition, the format of the investigation favoured an overrepresentation of cases from large clusters (which were more thoroughly investigated and more frequently sequenced). Such clusters might bias the profile of investigated cases compared with the general population. In our study, this translated with the overrepresentation of children and older people, with the latter causing an over-estimation of the hospitalisation rate. Immune status (low vaccine coverage in children and waning immunity in those aged above 60 years) could also play a role in the overrepresentation of these two groups.

## Conclusion

This study provides a thorough description of the B.1.640 variant circulation in France and additional information on case characteristics. Our findings illustrate how the combination of different surveillance systems and the collaboration of different public health actors can efficiently and reactively produce ‘data for action’ in the context of emerging SARS-CoV-2 variants. The B.1.640 variant circulated at a low level during the Delta period (July–December 2021) but disappeared with the emergence of Omicron (end of 2021), suggesting that its competitiveness in the French population was similar to Delta but lower than Omicron. Additional work using genomic data, in vitro studies and/or epidemiological analyses would be needed to better understand the dynamics and characteristics of B.1.640 compared with other circulating variants.

## Supplementary Data

425000 Supplement
