# Supplementary material for: Emergence, spread and characterisation of the SARS-CoV-2 variant B.1.640 circulating in France, October 2021 to February 2022
Source: Euro Surveill. 2023 Jun 1;28(22):2200671. doi: 10.2807/1560-7917.ES.2023.28.22.2200671 (PMC10236926; doi:10.2807/1560-7917.ES.2023.28.22.2200671)

Supplementary material

This supplementary material is hosted by Eurosurveillance as supporting information alongside the article **Emergence, spread and characterisation of the SARS-CoV-2 variant B.1.640 in France, October 2021 to February 2022** on behalf of the authors who remain responsible for the accuracy and appropriateness of the content. The same standards for ethics, copyright, attributions and permissions as for the article apply. Supplements are not edited by Eurosurveillance and the journal is not responsible for the maintenance of any links or email addresses provided therein.

S1. Time distribution of B.1.640 sequences (total and from representative surveillance) and B.1.640 cases that were investigated, by sampling week. Omicron cases that were previously investigated are added as reference.

**Sampling week**	**All sequences**		**Representative sequencing**		**Investigated B.1.640 cases**		**Investigated Omicron cases**	
	**N**	**%**	**N**	**%**	**N**	**%**	**N**	**%**
2021-W40	2	0.2	0	0	0	0	0	0
2021-W41	4	0.4	1	0.6	8	3	0	0
2021-W42	9	0.9	2	1.1	11	4.1	0	0
2021-W43	17	1.7	11	6.1	15	5.6	0	0
2021-W44	24	2.4	18	10.1	17	6.4	1	0.2
2021-W45	17	1.7	10	5.6	8	3	0	0
2021-W46	34	3.4	9	5	20	7.5	2	0.5
2021-W47	138	13.7	19	10.6	83	31.1	17	4.1
2021-W48	233	23.1	27	15.1	53	19.9	115	27.9
2021-W49	247	24.5	26	14.5	21	7.9	170	41.3
2021-W50	177	17.5	27	15.1	19	7.1	77	18.7
2021-W51	40	4	8	4.5	4	1.5	10	2.4
2021-W52	32	3.2	15	8.4	5	1.9	20	4.9
2022-W01	20	2	3	1.7	0	0	0	0
2022-W02	9	0.9	3	1.7	3	1.1	0	0
2022-W03	2	0.2	0	0	0	0	0	0
2022-W04	2	0.2	0	0	0	0	0	0
2022-W06	2	0.2	0	0	0	0	0	0
**Total**	**1009**	**100**	**179**	**100**	**267**	**100**	**412**	**100**

Supplementary material

S2. Comparison of investigated B.1.640 (N=272) and Omicron (N=468) cases.

variable	B.1.640			Omicron			p_{value} Chi²
	N	Total	%	N	Total	%	
Female	101	205	49.3	196	358	54.7	0.244
0-9	16	263	6.1	11	446	2.5	0.0259
10-14	21	263	8.0	17	446	3.8	0.0271
15-19	5	263	1.9	25	446	5.6	0.0297
20-29	35	263	13.3	113	446	25.3	0.0002
30-39	36	263	13.7	109	446	24.4	0.0009
40-49	49	263	18.6	67	446	15.0	0.2503
50-59	30	263	11.4	63	446	14.1	0.3572
60-69	26	263	9.9	29	446	6.5	0.1384
70-79	30	263	11.4	9	446	2.0	<0.0001
>=80	15	263	5.7	3	446	0.7	0.0001
Symptomatic infection	207	240	86.2	376	422	89.1	0.3358
Risk factors	29	116	25.0	45	284	15.8	0.0457
Hospitalization	19	226	8.4	7	340	2.1	0.0009
Intensive care	5	222	2.3	0	338	0	0.0208
Previous SARS-CoV-2 infection	4	102	3.9	39	279	14.0	0.0103
Unvaccinated	66	217	30.4	113	412	27.4	0.4862
Vaccinated >=2 doses	145	217	66.8	279	412	67.7	0.8895
Vaccinated 2 doses	126	198	63.6	251	384	65.4	0.7475
Vaccinated 3 doses	19	217	8.8	28	412	6.8	0.4660

Number (N) and percentage of cases with a given characteristic and total number of cases with available data for this variable (Total) are indicated.

Chi² tests were performed to compare B.1.640 and Omicron cases.

Significant differences are highlighted in light yellow ($p<0.05$) and dark yellow ($p<0.01$).

Supplementary material

S3. Comparison of hospitalised (N=19) and non-hospitalised (N=207) cases among investigated B.1.640 cases (N=272).

variable	Hospitalised			Not hospitalised			p_{value} Chi²
	N	Total	%	N	Total	%	
Female	6	14	42.9	80	158	50.6	0.7804
0-9	0	18	0.0	11	200	5.5	0.6463
10-14	0	18	0.0	16	200	8.0	0.4385
15-19	0	18	0.0	5	200	2.5	1
20-29	0	18	0.0	34	200	17.0	0.1176
30-39	0	18	0.0	31	200	15.5	0.1467
40-49	4	18	22.2	34	200	17.0	0.8142
50-59	1	18	5.6	24	200	12.0	0.6630
60-69	3	18	16.7	20	200	10.0	0.6303
70-79	2	18	11.1	19	200	9.5	1
>=80	8	18	44.4	6	200	3.0	<0.0001
>40ans	18	18	100.0	103	200	51.5	0.0002
>60ans	13	18	72.2	45	200	22.5	<0.0001
>70ans	10	18	55.6	25	200	12.5	<0.0001
>80ans	8	18	44.4	6	200	3.0	<0.0001
Symptomatic infection	19	19	100.0	174	207	84.1	0.1226
Risk factors	5	7	71.4	23	103	22.3	0.0148
Previous SARS-CoV-2 infection	19	19	100.0	0	207	0.0	<0.0001
Unvaccinated	5	10	50.0	0	206	0.0	<0.0001
Vaccinated >=2 doses	8	14	57.1	128	162	79.0	0.1233
Vaccinated 2 doses	7	13	53.8	110	144	76.4	0.1459
Vaccinated 3 doses	1	14	7.1	18	162	11.1	0.9919

Number (N) and percentage of cases with a given characteristic and total number of cases with available data for this variable (Total) are indicated. Chi² tests were performed to compare hospitalised and non-hospitalised cases. Significant differences are highlighted in light yellow (p<0.05) and dark yellow (p< 0.01).

S4. Coefficients of the Poisson regression model used to assess factors associated with hospitalization.

Variable	Levels	adjusted risk ratio	95% confidence interval	p value
Variant	B.1.640<br>(*vs* Omicron)	2.35	0.671 - 9.28	0.189
Vaccine status	2 or 3 doses<br>(*vs* 0 or 1 dose)	0.255	0.096 - 0.686	0.0055
Age	≥ 80<br>(*vs* < 80)	20.0	6.74 - 63.1	<0.0001
Intercept		0.0329	0.00931 - 0.0852	<0.0001

Figure S1. Age distribution of investigated cases infected by B.1.640 depending on whether they belonged to an important cluster or not.

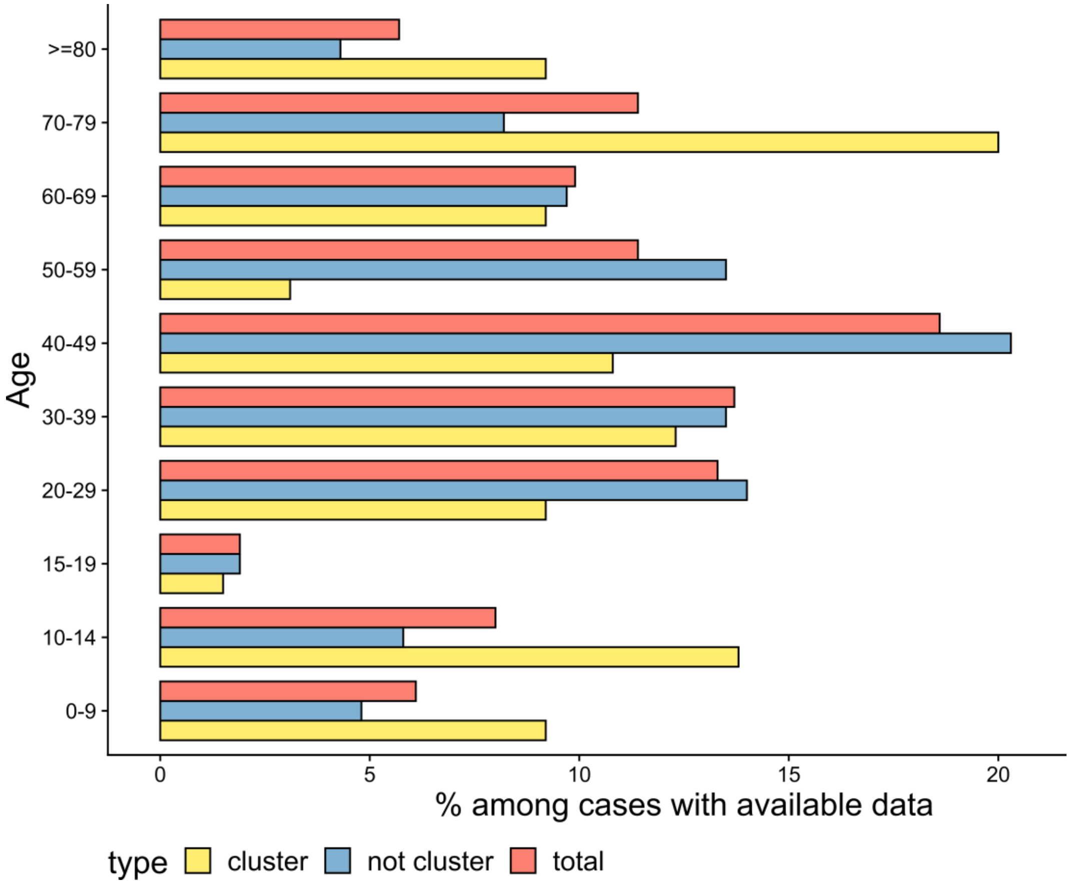

Supplement: Supplement [file 22-00671_SCHAEFFER_SUPPLEMENT.pdf]
